# Assessment of individual cognitive changes after deep brain stimulation surgery in Parkinson’s disease using the Neuropsychological Test Battery Vienna short version

**DOI:** 10.1007/s00508-017-1169-z

**Published:** 2017-02-07

**Authors:** Thomas Foki, Daniela Hitzl, Walter Pirker, Klaus Novak, Gisela Pusswald, Eduard Auff, Johann Lehrner

**Affiliations:** 1 Department of Neurology, Medical University of Vienna, Währinger Gürtel 18–20, 1097 Vienna, Austria; 2 Department of Neurology, Wilhelminenspital Wien, Vienna, Austria; 3 Department of Neurosurgery, Medical University of Vienna, Vienna, Austria

**Keywords:** Parkinson’s disease, Deep brain stimulation, Cognition, Subthalamic nucleus, MCI

## Abstract

Long-term therapy of Parkinson’s disease with L‑DOPA is associated with a high risk of developing motor fluctuations and dyskinesia. Deep brain stimulation (DBS) of the subthalamic nucleus (STN) can improve these motor complications. Although the positive effect on motor symptoms has been proven, postoperative cognitive decline has been documented. To tackle the impact of DBS on cognition, 18 DBS patients were compared to 25 best medically treated Parkinson’s patients, 24 patients with mild cognitive impairment (MCI) and 12 healthy controls using the Neuropsychological Test Battery Vienna short version (NTBV-short) for cognitive outcome 12 months after the first examination. Reliable change index methodology was used. Roughly 10% of DBS patients showed cognitive decline mainly affecting the domains attention and executive functioning (phonemic fluency). Further research is needed to identify the mechanisms that lead to improvement or deterioration of cognitive functions in individual cases.

## Introduction

Parkinson’s disease (PD) has traditionally been regarded as a movement disorder characterized by bradykinesia, rigidity and tremor. In recent years, it has been recognized that the clinical spectrum of PD extends beyond the pure motor syndrome and includes a plethora of autonomic, sensory, neuropsychiatric and cognitive features. Impaired cognitive performance in domains such as attention, language, memory and executive functions has been documented in a number of studies [1, 2] and PD patients also have a higher risk of developing dementia [3].

Although sufficient control of motor symptoms can be attained with pharmacological treatment at the beginning of the disease, barely controllable motor complications develop over the long term. In cases with refractory motor complications, deep brain stimulation (DBS) offers an alternative to oral drug treatment and can lead to a marked improvement of these motor complications and of quality of life [4]. Although DBS is an established surgical therapy of motor symptoms in PD, postoperative cognitive dysfunction has been reported [5–7]. In general, it was found that DBS compromises executive functions, especially verbal fluency [5, 8]. Results concerning other neuropsychological tasks show heterogeneous findings. While some studies found additional worsening in working memory, attention and memory [8], others described changes in speed of mental processing, set switching and phonemic fluency [9].

Reliable change index (RCI) methodology was developed to take into account variances in test scores taken at two times [11]. RCI methodology is based on test-retest reliability (rtt) of tests and is a method to investigate outcome effects in single patients. The RCI methodology aims to record changes in results at two times of measurement without being affected by chance. Changes of outcome results due to practice effects have to be differentiated from changes due to disease effects or intervention effects. In serial testing procedures improvement in test performance can be achieved solely through practice effects. On the other hand, effects of aging and disease progression may influence test outcome. Furthermore, effects of therapeutic interventions must be considered. Because of the neurodegenerative nature of PD, progressive changes in neuropsychological testing in individual cases over time have to be expected. Additionally, changes due to clinical and therapeutic effects must be considered, e. g. medication effects, related fluctuations and depressive symptoms. To take these factors into account, RCI methodology has been developed. There are several reports available which describe RCI methodology in PD [10, 11]. Recent studies have investigated the effect of DBS on cognition in single patients using RCI methodology. In general, significant cognitive deterioration was found only in a few subthalamic nucleus (STN)-DBS patients suggesting that neuropsychological evaluations may identify possible mild cognitive changes following surgery [12–19]. An open question is whether cognitive changes in after DBS in PD patients using RCI methodology is comparable to changes in healthy age matched controls controlling for aging effects and other clinical populations such as mild cognitive impairment (MCI) controlling for neurodegenerative effects.

The purpose of this study was to investigate the impact of DBS on cognition using RCI methodology. We wanted to tackle changes of neurocognitive functioning related to treatment in PD patients who underwent surgery compared to best medically treated PD patients. Furthermore, an important goal was to study RCI methodology in healthy controls and patients with MCI and compare it to PD patients with surgery.

## Patients and methods

The current data are part of a larger research project, the Vienna Mild Cognitive Impairment and Cognitive Decline in Parkinson’s Disease study (VMCI-CD-PD study). The VMCI-CD-PD study is a prospective cohort study including consecutive, community-dwelling PD patients who attend the movement disorder clinic for assessment of parkinsonism. The study protocol was in accordance with the Helsinki Declaration and approved by the ethical committee of the Medical University of Vienna.

All PD patients underwent clinical examination and neuropsychological testing. The clinical assessment encompassed a complete medical history, a detailed history of PD, which was obtained using a standardized interview, and a complete neurological examination including the motor section of the Unified Parkinson’s Disease Rating Scale (UPDRS-III) [20] and the modified Hoehn and Yahr scale [21]. Clinical examination and neuropsychological testing were performed during the “on” state. Patients who had never undergone computed tomography (CT) or magnetic resonance imaging (MRI) during the course of PD and patients showing clinical features incompatible with previous imaging results were referred to structural imaging. Both neuroimaging and clinical features were used to determine significant cerebrovascular disease or other comorbid conditions with a potential impact on cognitive outcomes.

Inclusion and exclusion criteria were similar to those used in other studies. All PD patients had to fulfill UK Parkinson’s Disease Society Brain Bank criteria [22] for probable PD. Inclusion and exclusion criteria were specified as follows: PD-DBS patients suffered from PD for at least 5 years with a positive response to L‑DOPA treatment. All PD-DBS patients suffered from motor complications (fluctuations with or without dyskinesia) refractory to oral drug treatment or treatment-resistant PD tremor. Patients with secondary or atypical parkinsonism were excluded. Severe cognitive impairments, such as dementia and uncontrolled psychiatric disorders also represented exclusion criteria. Comorbidities and structural brain lesions that would interfere with the surgical procedure were ruled out. Controls and patients were excluded from the study if any of the following conditions applied: (a) evidence of a past stroke as determined by neuroradiological and clinical examination, (b) history of severe head injury, (c) current psychiatric diagnosis according to ICD-10 with the exception of patients with (sub)depressive symptoms, (d) any medical condition that can lead to cognitive deterioration including renal, respiratory, cardiac and hepatic disease, or (e) a diagnosis of dementia according to DSM IV criteria [23]. Patients were assessed on their regular medication and were required to have a mini-mental state examination (MMSE) score of ≥ 26.

All participants were subjected to the short version of the Neuropsychological Test battery Vienna (NTBV) for assessment of their neurocognitive functions. The NTBV-short assesses several cognitive domains including attention, executive functioning, language and memory domains with corresponding domain z scores [24]. In addition, a total z‑score across all tests was computed [25]. The “Alters-Konzentrations-Test” (AKT) [26] and the symbol counting task from the cerebral insufficiency test (CI) [27] were used to assess attention. Executive functions were investigated using the Trail Making Test A, the Maze Test from the NAI test battery [28], and the interference test from the CI [27]. Naming as many words beginning with the letter f that came to mind within 1 min was used to tap lexical verbal fluency [29]. In order to test language functions, we used a verbal fluency task naming as many animals [29], and a confrontation naming task (Boston Naming Test) [30]. Episodic memory was tested using the Verbal Selective Reminding Test (VSRT) with the subtests of immediate recall, total recall, delayed recall and recognition [31]. After the completion of the tests, the cognitive status was determined according to the Petersen criteria [32], and the cut-off score used was 1.5 standard deviations below age and education corrected norms using a normative sample of cognitively healthy controls. For this purpose, the flexible Generalized Additive Models for Location, Scale and Shape (GAMLSS) model class was used [33]. The NTBV was performed two times, the second testing was performed 1 year after the first.

The study included 79 participants subdivided into 4 groups. Patients with PD were divided into a PD-DBS group (DBS plus best medical treatment) and a PD-BMT group (best medical treatment only). The DBS group underwent deep brain stimulation after the first testing. Patients received bilateral magnetic resonance (MR)-based, stereotactic DBS surgery. The optimum electrode position within the STN was assessed intraoperatively in the awake patient, applying macrostimulation to test for motor improvement and side effects. A small minority of PD patients with anxiety disorder underwent DBS surgery under general anesthesia. Postoperative MR imaging (MRI) was used to exclude perioperative structural lesions and to reassure correct electrode position. Starting with standard DBS parameters (60 μs impulse duration at 130 Hz), the voltage was gradually increased over 1–2 weeks and stimulation parameters adjusted individually. In parallel, dopaminergic therapy was decreased gradually [34, 35]. Community-dwelling patients complaining of cognitive problems who came to the memory outpatient clinic for assessment of a possible cognitive disorder were included in the study. Mild cognitive impairment (MCI) was defined according to the Petersen criteria [32].

The healthy control group consisted of patients without PD and cognitive impairments. Great care was taken to enrol a sufficient number of cognitively healthy control subjects living independently at home. Control subjects were recruited by means of advertisements and underwent a rigorous screening evaluation using a standardized clinical interview and cognitive screening. Imaging procedures, neurological examination, standard laboratory blood tests and informant reports were not included in the evaluation. They were assessed as being in good health. Criteria for healthy function were identified as being similar to those in the Mayo research studies [36]: (a) no active neurological or psychiatric disease, (b) no psychotropic medications, and (c) the subjects may have medical disorders but neither they nor their treatment compromises cognitive function. Cognitive status was given special attention and cognitively healthy control subjects were screened for intact cognition. They were required to have an MMSE score greater than or equal to 27 and a MOCA score greater than or equal to 26 adjusted for education. Control subjects did not overtly complain about cognitive problems.

All four patient groups were matched for age, gender, MMSE status [37], verbal intelligence (WST) [38] and depressive symptoms (BDI-II) [39]. Using Kruskal-Wallis analyses no statistical group differences were found (all *p* > 0.3). Demographic and clinical data are shown in Table 1.

**Table 1:** Demographic and clinical data

	Total group (*N* = 79)	Healthy control (*N* = 12)	MCI (*N* = 24)	PD-BMT (*N* = 25)	PD-DBS (*N* = 18)
Age	62.9 ± 7.7	65.1 ± 5.7	63.1 ± 7.4	62.9 ± 6.5	60.0 ± 10.2
Education	10.1 ± 2.3	9.9 ± 2.3	10.0 ± 2.3	10.4 ± 2.35	10.1 ± 2.3
Sex (m/w)	37/42	3/9	14/10	14/11	6/12
MMSE	28.3 ± 1.3	28.8 ± 0.8	28.1 ± 1.2	28.4 ± 1.2	28.0 ± 1.6
WST	103.9 ± 10.0	103.0 ± 7.5	104.5 ± 9.9	103.2 ± 10.6	106.1 ± 12.1
BDI-II	10.2 ± 6.0	10.7 ± 5.8	10.3 ± 5.4	9.3 ± 6.1	11.7 ± 7.3
UPDRS motor score	–	–	–	28.4 ± 12.7	25.1 ± 10.5
Hoehn & Yahr scale	–	–	–	3.68 ± 1.9	3.8 ± 1.0

*MMSE* mini mental state examination, *WST* “Wortschatz Test”, *BDI-II* Beck-Depression Inventory, *UPDRS* Unified Parkinson’s Disease Rating Scale

## Statistical analyses

Based on the mean test z‑scores for each cognitive domain, standard deviations were calculated for each domain separated by group [10, 11]. Mean test z‑scores and standard deviations were used for calculating the RCI as follows:

$$RCI=\left (\left (X_{2}-X_{1}\right )-\left (M_{2}-M_{1}\right )\right )\cdot SED$$

The difference of (X2-X1) characterizes the individual test scores of a participant at the two test sessions over time. M2 and M1 represent the mean z‑scores of the group the participant is compared to. The calculation of the standard error of difference (SED) uses the standard deviation and rtt of the specific comparison group. The standard deviation is taken from the first test session. The rtt coefficient of a given test is calculated by using test scores of the first test session (x1) and test scores of the second test session (x2).

$$SED=\sqrt{2\cdot SEM}$$

$$SEM=SD\sqrt{\left (1-r_{tt}\right )}$$

This formula is based on Chelune et al. [11] and a significant change can be assumed at a change of ±1.64 (*p* = 0.05). Based on this a confidence interval is calculated to determine a minimum and a maximum limit which represent significant changes.

In order to ensure reproducibility for future studies, the calculations are shown here using a case example. A patient taken from the DBS group is compared to the PD-BMT group for the memory domain. All scores have already been transformed into z‑scores. The calculation process is divided into two steps. At first, calculation of the confidence interval using mean score and standard deviation of the PD-BMT group is performed for both test sessions. Mean score of the memory domain at first testing session was −0.188 with a standard deviation of 0.538 for the PD group. Mean score of the memory domain at the second testing session was −0.472 with a standard deviation of 0.671 for the PD-BMT group. The rtt coefficient (rtt = 0.649) results from the correlation of the test scores across time for the PD group. The formula for the standard error of measurement (SEM) is derived by including the test-retest correlation coefficient and standard deviation from the PD-BMT group.

$$SEM=0,538\cdot\sqrt{\left (1-0,649\right )}=0,319$$

Based on the standard error of measurement the SED is calculated.

$$SED=\sqrt{2\cdot 0,319}=0,451$$

According to Chelune et al. [11] a deviation of ±1.64 can be assumed as statistically significant.

$$CI=1,64\cdot0,451=0,74$$

The upper and lower scores of the confidence interval are serving as limiting values for defining statistical significance. After subtracting the second and the first mean score (M2–M1), the confidence interval is added or subtracted. Difference of mean scores (DM): M2–M1 = −0.284. Limiting value deterioration: −CI + DM = −0.74 − 0.284 = −1.024. Limiting value improvement: CI + DM = 0.74 − 0.284 = 0.456. A patient’s RCI has to be higher or lower than the limiting values in order to detect a statistically significant change between the first and second testing. The scores of the reliability coefficient, standard error of the mean and SED are calculated like shown above. For the memory domain the figures are as follows: rtt = 0.577; SEM = 0.621; SED = 0.878; M1 = −0.456; M2 = −0.635.

The patient chosen for the illustrative example has achieved a z-score of −2.4 (X1) in the first and −1.9 (X2) in the second testing in the memory domain. The mean scores of the group and the individual test scores are put in the formula for calculating the reliable change index: RCI = (((−1.9) − (−2.4)) − ((−0.635) − (−0.456)))∙0.878 = 0.596. This results in a RCI score of 0.596 for this patient. This value is compared with the limiting values calculated above. The upper limiting value of 0.456 is surpassed; therefore, an improvement of memory ability in this patient in comparison to the Parkinson’s disease group has been detected.

## Results

In order to determine the individual performance using RCI methodology, RCI calculations were performed for each participant in each group for every domain. The scores of the reliability coefficient, SEM, SED and the limiting values for improvement and deterioration as calculated for the PD-MBT group, healthy control group and MCI group and are shown in Table 2. The table shows the results for the RCI analysis comparing the DBS group to the calculated limiting values of the PD-BMT group, the control group and the MCI group.

**Table 2:** The rtt, SEM, SED and limiting values for the PD-BMT group, control group and MCI group

	Mean score and SD 1st testing	Mean score and SD 2nd testing	rtt	SEM	SED	Limiting value deterioration	Limiting value improvement
*PD-BMT group*							
Attention	−0.7 ± 1.4	−1.0 ± 1.2	0.7	0.7	0.9	−1.8	1.1
Language	−0.5 ± 0.7	−0.5 ± 0.8	0.6	0.5	0.6	−1.1	1.0
Executive function (phonemic fluency)	−0.5 ± 1.0	−0.4 ± 1.1	0.7	0.5	0.7	−1.1	1.3
Memory	−0.2 ± 0.5	−0.5 ± 0.6	0.6	0.3	0.5	−1.0	0.4
Executive function (nonverbal planning)	−0.8 ± 1.1	−0.6 ± 1.0	0.6	0.7	1.0	−1.5	1.7
Total NTBV short	−0.5 ± 0.7	−0.6 ± 0.6	0.8	0.3	0.5	−0.8	0.6
*Control group*							
Attention	0.8 ± 1.2	0.2 ± 0.9	0.3	0.9	1.3	−2.6	1.6
Language	−0.1 ± 0.5	−0.2 ± 0.6	0.5	0.3	0.5	−0.8	1.1
Executive function (phonemic fluency)	0.1 ± 0.9	0.2 ± 1.1	0.6	0.6	0.9	−1.2	1.5
Memory	−0.1 ± 0.7	−0.3 ± 0.5	0.2	0.6	0.8	−1.7	1.0
Executive function (nonverbal planning)	0.6 ± 0.8	0.7 ± 0.7	0.5	0.5	0.8	−1.0	1.4
Total NTBV short	0.3 ± 0.5	0.2 ± 0.5	0.5	0.3	0.4	−0.7	0.6
*MCI group*							
Attention	−0.2 ± 1.3	−0.1 ± 1.3	0.6	0.7	1.0	−1.6	1.7
Language	−0.6 ± 0.8	−0.7 ± 0.9	0.6	0.5	0.6	−1.1	0.9
Executive function (phonemic fluency)	−0.6 ± 1.2	−0.5 ± 1.0	0.6	0.8	1.1	−1.8	1.8
Memory	−0.5 ± 0.9	−1.0 ± 1.1	0.6	0.5	0.7	−1.6	0.6
Executive function (nonverbal planning)	0.1 ± 1.1	−0.1 ± 1.2	0.7	0.6	0.8	−1.5	1.0
Total NTBV short	−0.3 ± 0.7	−0.4 ± 0.8	0.6	0.4	0.6	−1.0	0.7

*rtt* test-retest reliability, *SEM* standard error of measurement, *SED* standard error of difference, *PD-BMT* Parkinson’s Disease best medically treated, *MCI* Mild cognitive impairment, *NTBV* Neuropsychological Test Battery Vienna

The results of the comparison between the PD-DBS group and all other groups are displayed in Table 3. The only significant deterioration detected can be found in the domain executive functioning (phonemic fluency) with 11.1% of patients showing a significant deterioration. The comparison of the PD-DBS group with the healthy control group revealed significant deteriorations in the domain attention as well as in the domain of executive functioning (phonemic fluency) in 11.1% of patients. No other domain showed significant deteriorations. When comparing MCI patients to DBS patients no significant deteriorations have been detected.

**Table 3:** Results of individual comparison between PD-BMT group–PD-DBS group, control group–PD-DBS group and MCI group–PD-DBS group

	*N* deteriorated	% deteriorated	*N* no change	% no change	*N* improved	% improved
*PD-BMT – PD-DBS*						
Attention	0	0.0	18	100	0	0.0
Language	0	0.0	18	100	0	0.0
Executive function (*phonemic fluency*)	2	11.1	14	77.7	2	11.1
Memory	0	0.0	15	83.3	3	16.6
Executive function (nonverbal planning)	0	0.0	18	100	0	0.0
Total NTBV short	0	0.0	18	100	0	0.0
*Control – PD-DBS*						
Attention	2	11.1	16	88.8	0	0.0
Language	0	0.0	18	100	0	0.0
Executive function (*phonemic fluency*)	2	11.1	14	77.7	2	11.1
Memory	0	0.0	17	94.4	1	5.5
Executive function (nonverbal planning)	0	0.0	18	100	0	0.0
Total NTBV short	0	0.0	18	100	0	0.0
*MCI – PD-DBS*						
*Attention*	0	0.0	18	100	0	0.0
*Language*	0	0.0	18	100	0	0.0
*Executive function (phonemic fluency)*	0	0.0	17	94.4	1	5.5
*Memory*	0	0.0	17	94.4	1	5.5
*Executive function (Nonverbal planning)*	0	0.0	18	100	0	0.0
*Total NTBV short*	0	0.0	18	100	0	0.0

*PD-DBS* Parkinson’s disease deep brain stimulation, *PD-BMT* Parkinson’s disease best medically treated, *MCI* Mild cognitive impairment, *NTBV* Neuropsychological Test Battery Vienna

## Discussion

Deep brain stimulation has evolved to become an important therapeutic option in the treatment of PD [4]. As DBS is a lifelong treatment, it is important to evaluate possible side effects, particularly cognitive decline after DBS treatment. The gold standard to evaluate cognitive outcome is randomized controlled studies; however, randomized controlled study methodology is not suitable for assessing cognitive outcome in a single patient in the clinical setting. In order to predict cognitive outcome in a single patient in clinical settings, RCI methodology has been developed [11]. Our study investigated cognitive outcome after DBS in comparison to three groups, namely a best medically treated PD group (PD-BMT group), a healthy control group without cognitive impairment (healthy control group) and a group consisting of patients having mild cognitive impairment (MCI group). All groups were matched in terms of age, education, IQ, MMSE status and depressive symptoms. When compared to a PD-BMT group we found that approximately 10% of patients showed cognitive deterioration 1 year after DBS. The cognitive domain only affected was executive functioning, namely phonemic fluency. All other domains showed no significant cognitive deterioration after DBS compared to a PD-BMT group. It is noteworthy that significant deterioration of a particular cognitive function only affected a small minority of our patient cohort.

Several previous studies found changes in phonemic fluency, mostly deteriorations [40–42]. The decline in phonemic fluency represents the most common neurocognitive change after DBS. It seems that DBS has a negative impact on this executive function at least in some patients. Studies on other neurosurgical interventions such as pallidotomy and pallidus pars interna (GPi) stimulation also reported deterioration in phonemic fluency [43–45] although to a lesser degree [7]. The deterioration of phonemic fluency may therefore be a side effect of frontal lobe trajectory use to access the basal ganglia [40].

We found no significant changes for the domain of language. Prior studies have shown positive changes after surgery on different levels of language and speech [46, 47]. These improvements have not been found in all studies and Funkiewiez et al. even found diminished verbal abilities [48]. Impairment in language may occur immediately after surgery caused by interference with neural networks. In long-term follow-up examinations no further deterioration was found after 3 years [49].

The memory domain showed no decline in our study. The fact that DBS may cause enhancement of memory abilities has been shown in previous studies [50, 51]. Direct and indirect projections of the STN to the dorsal striatum may be implicated in memory functions. Memory improvements after DBS may be explained by an improved striatal transmission of dopamine [40–42].

The results of our study corroborate previous studies investigating the cognitive outcome after DBS in PD using RCI methodology. As in our study, earlier studies also found low prevalence of cognitive decline in single cognitive domains [12–19].

Several authors discussed the influence on cognitive results by the neurosurgical intervention itself. As cerebral tissue may be aggrieved during surgery, pathways may be impaired without interference caused by stimulation. Prior studies also implied that postoperative outcome is affected by factors other than surgery and stimulation. Potential negative predispositions are high age when receiving stimulation and a low number of years of education [43–45].

An important task of the present study was to compare DBS patients to a healthy control group and a group with MCI controlling for aging effects and effects of cognitive deterioration besides PD. When compared to the healthy control group we found a significant decline in attention in roughly 10% of DBS patients showing that there is a mild decline in the domain of attention above that seen in normal aging. Comparing PD-DBS patients and MCI patients no significant cognitive decline could be detected indicating that the DBS procedure did not produce significant cognitive changes above that seen in patients with mild cognitive impairment. To our knowledge, this is the first study to report such findings.

As with every other study this study has strengths and limitations. Using RCI methodology is a strength enabling direct comparison of different groups in single patient analysis. The PD patients and controls groups were closely matched in terms of age, education, IQ, MMSE status and depressive symptoms. A limitation is certainly the partially small group sizes. Another limitation is that the findings may not be generalized to all PD patients because our patients were recruited in a university-based specialized clinic for movement disorders.

Assessment of intraindividual cognitive stability and change is of considerable importance for the diagnosis of incident dementia and evaluation of treatment effectiveness in several clinical populations e. g. including PD and Alzheimer’s Disease (AD). We provide rtt reliability scores, expected practice effects and practice-corrected reliable change indices for the NTBV. Our data may be useful to researchers and clinicians interested in determining the statistical significance of change in cognitive test performance in patients with PD over a time interval of 12 months.

Although some parts of cognition showed improvement as well as deterioration, most PD patients subjectively do not suffer from these changes in terms of activities of daily living [48]. In general, DBS represents a safe and effective PD therapy when motor complications begin to decrease quality of life. Side effects of the treatment are often slight, intermittent and well-tolerated by patients. The advantages of the therapy are usually predominant. Future studies should also assess the impact of DBS on individual patient psychosocial outcome.

## References

[1] Hobson P, Meara J (2015). Mild cognitive impairment in Parkinson’s disease and its progression onto dementia: A 16-year outcome evaluation of the Denbighshire cohort. Int J Ger Psychiat.

[2] Lehrner J, Zach H, Moser D (2014). Prevalence of mild cognitive impairment subtypes in patients with parkinson’s disease – comparison of two modes of classification. Z Neuropsychol.

[3] Garcia-Ptacek S, Kramberger M (2016). Parkinson disease and dementia. J Geriat Psychiatneurol.

[4] Hickey P, Stacy M (2016). Deep brain stimulation: A paradigm shifting approach to treat parkinson’s disease. Front Neurosci.

[5] Bordini BJ, Garg A, Gallagher CL, Bell B, Garell PC (2007). Neuropsychological effects of bilateral deep brain stimulation of the subthalamic nucleus in Parkinson’s disease. Stereotact Funct Neurosurg.

[6] Altug FA, Acar G, Cavlak U (2011). The influence of Subthalamic nucleus deep brain stimulation on physical, emotional, cognitive functions and daily living activities in patients with Parkinson’s disease. Turk Neurosurg.

[7] Combs HL, Folley BS, Berry DTR (2015). Cognition and depression following deep brain stimulation of the Subthalamic nucleus and Globus Pallidus pars Internus in Parkinson’s disease: A Meta-analysis. Neuropsychol Rev.

[8] Halpern CH, Rick JH, Danish SF, Grossman M, Baltuch GH (2009). Cognition following bilateral deep brain stimulation surgery of the subthalamic nucleus for Parkinson’s disease. Int J Geriatr Psychiat.

[9] Saint-Cyr JA, Trepanier LL, Kumar R, Lozano AM, Lang AE (2000). Neuropsychological consequences of chronic bilateral stimulation of the subthalamic nucleus in Parkinson’s disease. Brain.

[10] Ringendahl H (2013). Neuropsychologische Verlaufsdiagnostik bei Parkinson-Patienten – Darstellung kritischer Test-Retestdifferenzen für die Einzelfalldiagnostik. Z Neuropsychol.

[11] Chelune GJ, Naugle RI, Lueders H, Sedlak J, Awad IA (1993). Individual change after epilepsy surgery: Practice effects and base-rate information. Neuropsychol.

[12] York MK, Dulay M, Macias A, Levin HS, Grossman R, Simpson R (2008). Cognitive declines following bilateral subthalamic nucleus deep brain stimulation for the treatment of Parkinson’s disease. J Neurol Neurosurg Psychiat.

[13] Schoenberg MR, Duff K, Mold J, Scott JG (2008). Evaluating change in Parkinson’s disease following STN DBS: Development of reliable change indices for the RBANS. Arch Clin Neuropsychol.

[14] Schoenberg MR, Rinehardt E, Duff K, Mattingly M, Bharucha KJ, Scott JG (2012). Assessing reliable change using the repeatable battery for the assessment of Neuropsychological status (RBANS) for patients with Parkinson’s disease undergoing deep brain stimulation (DBS) surgery. Clin Neuropsychol.

[15] Williams AE, Arzola GM, Strutt AM, Simpson R, Jankovic J, York MK (2011). Cognitive outcome and reliable change indices two years following bilateral subthalamic nucleus deep brain stimulation. Park Rel Dis.

[16] Mikos A, Zahodne L, Okun MS, Foote K, Bowers D (2010). Cognitive declines after unilateral deep brain stimulation surgery in Parkinson’s disease: A controlled study using reliable change, part II. Clin Neuropsychol.

[17] Rinehardt E, Duff K, Schoenberg M, Mattingly M, Bharucha K, Scott J (2010). Cognitive change on the repeatable battery of neuropsychological status (RBANS) in parkinson’s disease with and without bilateral subthalamic nucleus deep brain stimulation surgery. Clin Neuropsychol.

[18] Zahodne LB, Okun MS, Foote KD (2009). Cognitive declines one year after unilateral deep brain stimulation surgery in parkinson’s disease: A controlled study using reliable change. Clin Neuropsychol.

[19] Higginson CI, Wheelock VL, Levine D, King DS, Pappas CTE, Sigvardt KA (2009). The clinical significance of neuropsychological changes following bilateral subthalamic nucleus deep brain stimulation for Parkinson’s disease. J Clin Exp Neuropsychol.

[20] Goetz CGF, Martinez-Martin P, Poewe W (2007). Movement Disorder Society-sponsored revision of the Unified Parkinson’s Disease Rating Scale (MDS-UPDRS): Process, format, and clinimetric testing plan. Mov Disord.

[21] Hoehn MM, Yahr MD (1967). Parkinsonism: Onset, progression and mortality. Neurol.

[22] Gibb WRL (1988). The relevance of the Lewy body to the pathogenesis of idiopathic Parkinson’s disease. J Neurol Neurosurg Psychiat.

[23] American Psychiatric Association (2013). Diagnostic and Statistical Manual of Mental Disorders (DSM).

[24] Lehrner J, Gleiß A, Maly J, Auff E, Dal-Bianco P (2007). Demenzdiagnostik mit Hilfe der Vienna Neuropsychologischen Testbatterie (VNTB): Standardisierung, Normierung und Validierung. Psychol Öst.

[25] Pusswald GM, Gleiß A, Janzek-Hawlat S, Auff E, Dal-Bianco P, Lehrner J (2013). Prevalence of mild cognitive impairment subtypes in patients attending a memory outpatient clinic – comparison of two models of mild cognitive impairment classificaiton. Results of the Vienna Conversion to Dementia Study. Alzheimers Dement.

[26] Gatterer G (1990). Alters-Konzentrations-Test (AKT).

[27] Lehrl S, Fischer B (1997). Kurztest für cerebrale Insuffizient (c.I.-Test).

[28] Oswald WD, Fleischmann UM (1995). Das Nürnberger-Alters-Inventar (NAI).

[29] Goodglass H, Kaplan P (1983). The assessment of the aphasia and related disorders.

[30] Morris JC, Heyman A, Mohs RC (1989). The Consortium to Establish a Registry for Alzheimer’s Disease (CERAD). Part I. Clinical and neuropsychological assessment of Alzheimer’s disease. Neurol.

[31] Lehrner J, Gleiß A, Maly J, Auff E, Dal-Bianco P (2006). Der Verbale Selektive Reminding Test (VSRT) Ein Verfahren zur Überprüfung verbaler Gedächtnisfunktionen. Neuropsychiatr.

[32] Petersen RC, Smith GE, Waring SC, Ivnik RJ, Tangalos EG, Kokmen E (1999). Mild cognitive impairment: clinical characterization and outcome. Arch Neurol.

[33] Stasinopoulus DM, Rigby RA (2007). Generalized additive models for location scale and shape (GAMLSS). J Stat Software.

[34] Deuschl G, Schade-Brittinger C, Krack P (2006). A randomized trial of deep-brain stimulation for Parkinson’s disease. N Engl J Med.

[35] Diener HCPN, Berlit P (2003). Leitlinien für Diagnostik und Therapie in der Neurologie.

[36] Ivnik R (1992). Mayo’s older Americans normative studies: WAIS-R, WMS-R and AVLT norms for ages 56 through 97. Clin Neuropsychol.

[37] Folstein MF, Folstein SE, McHugh PR (1975). “Mini-mental state”. A practical method for grading the cognitive state of patients for the clinician. J Psychiatr Res.

[38] Schmidt K‑HM P (1992). Wortschatztest (WST).

[39] Hautzinger MB, Worall H, Keller F (1995). Beck Depressions-Inventar (BDI-II). Testhandbuch.

[40] Schultz W (1982). Depletion of dopamine in the striatum as an experimental model of Parkinsonism: direct effects and adaptive mechanisms. Prog Neurobiol.

[41] Parent A, Smith Y (1987). Organization of efferent projections of the subthalamic nucleus in the squirrel monkey as revealed by retrograde labeling methods. Brain Res.

[42] Squire LR, Zola SM (1996). Structure and function of declarative and nondeclarative memory systems. Proc Natl Acad Sci USA.

[43] Limousin P, Greene J, Pollak P, Rothwell J, Benabid AL, Frackowiak R (1997). Changes in cerebral activity pattern due to subthalamic nucleus or internal pallidum stimulation in Parkinson’s disease. Ann Neurol.

[44] Alegret M, Junque C, Valldeoriola F (2001). Effects of bilateral subthalamic stimulation on cognitive function in Parkinson disease. Arch Neurol.

[45] McCarter RJ, Walton NH, Rowan AF, Gill SS, Palomo M (2000). Cognitive functioning after subthalamic nucleotomy for refractory Parkinson’s disease. J Neurol Neurosurg Psychiat.

[46] Whelan BM, Murdoch BE, Theodoros DG, Hall B, Silburn P (2003). Defining a role for the subthalamic nucleus within operative theoretical models of subcortical participation in language. J Neurol Neurosurg Psychiat.

[47] Moretti R, Torre P, Antonello RM (2003). Neuropsychological changes after subthalamic nucleus stimulation: a 12 month follow-up in nine patients with Parkinson’s disease. Park Relat Disord.

[48] Funkiewiez A, Ardouin C, Caputo E (2004). Long term effects of bilateral subthalamic nucleus stimulation on cognitive function, mood, and behaviour in Parkinson’s disease. J Neurol Neurosurg Psychiat.

[49] Benazzouz A (2006). High-frequency stimulation of the Subthalamic nucleus in Parkinson’s disease. Eur Neurol Rev.

[50] Halbig TD, Gruber D, Kopp UA (2004). Subthalamic stimulation differentially modulates declarative and nondeclarative memory. Neurorep..

[51] Hilker R, Voges J, Weisenbach S (2004). Subthalamic nucleus stimulation restores glucose metabolism in associative and limbic cortices and in cerebellum: Evidence from a FDG-PET study in advanced Parkinson’s disease. J Cereb Blood Flow Metab.

